# Notch Signaling Pathway Promotes Th17 Cell Differentiation and Participates in Thyroid Autoimmune Injury in Experimental Autoimmune Thyroiditis Mice

**DOI:** 10.1155/2023/1195149

**Published:** 2023-01-06

**Authors:** Hao Liu, Yiwen Li, Yujiao Zhu, Lei Ma, Haibo Xue

**Affiliations:** ^1^Department of Endocrinology and Metabolism, Binzhou Medical University Hospital, No. 661 Second Huanghe Road, Binzhou 256603, China; ^2^Department of Dermatology, Binzhou Medical University Hospital, No. 661 Second Huanghe Road, Binzhou 256603, China

## Abstract

**Purpose:**

To investigate whether the Notch signaling pathway participates in the occurrence and development of experimental autoimmune thyroiditis (EAT) by affecting the differentiation and function of Th17 cells.

**Materials and Methods:**

Experimental mice were randomly divided into a control group, an EAT-A group (porcine thyroid immunoglobulin- (pTg-) treated mice) and an EAT-B group (treated with the DAPT *γ*-secretase inhibitor before pTg). HE staining, IHC staining, flow cytometry, RT-qPCR, and ELISA were used to evaluate the degrees of thyroiditis, detect the percentage of Th17 cells and measure the expression of retinoic acid-related orphan receptor gamma t (ROR*γ*t), interleukin-17A (IL-17A), and the main components of the Notch signaling pathway.

**Results:**

The degrees of thyroiditis, the proportions of Th17 cells, and the expression of ROR*γ*t and IL-17A were significantly decreased in the EAT-B group after blocking the Notch signaling pathway by DAPT, and these parameters were significantly increased in the EAT-A group compared to the control group (all *P* < 0.05). Additionally, the Th17 cell percentages and IL-17A concentrations in spleen mononuclear cells (SMCs) from EAT-A mice decreased in a dose-dependent manner after DAPT treatment in vitro (all *P* < 0.01). Correlation analyses revealed that the Th17 cell percentages were positively correlated with the serum TgAb titers, Notch pathway-related mRNA expression levels, and IL-17A concentrations in EAT mice (all *P* < 0.05).

**Conclusions:**

The expression of Notch signaling pathway components was upregulated in EAT mice, but blockade of the Notch signaling pathway alleviated the degree of thyroiditis, decreased the Th17 cell proportions, and downregulated the IL-17A effector cytokine both in vivo and in vitro. These findings suggested that the Notch signaling pathway may be involved in the pathogenesis of thyroid autoimmune injury in EAT mice by promoting the differentiation of Th17 cells.

## 1. Introduction

Autoimmune thyroiditis (AIT) is one of the most common thyroid diseases, and its typical pathological characteristics are infiltration of lymphocytes within the thyroid, which leads to thyroid follicular cell atrophy and fibrosis as well as the destruction of epithelial cells, which is accompanied by increased organ-specific autoimmune antibodies, namely, thyroglobulin antibody (TgAb) and thyroid peroxidase antibody (TPOAb) [1]. AIT progresses naturally to hypothyroidism, but there are no specific prevention and treatment measures at present. The thyroid disease, iodine nutrition, and diabetes epidemiology (TIDE) survey has reported that the overall positive rate of thyroid autoimmune antibodies is 14.19% among Chinese adults, and the positive rates of TPOAb and TgAb are 10.19% and 9.70%, respectively [2]. Therefore, it is clinically important to clarify the pathogenesis of AIT for its effective prevention and treatment.

During AIT, the thyroid is mainly infiltrated by T cells, and autoimmune injury mediated by T cells is the key pathogenesis. However, the exact mechanism has not yet been elucidated. As a subgroup of CD4^+^ T cells capable of secreting interleukin-17 (IL-17), Th17 cells play an important role in both autoimmune diseases and antibody defense response [3]. Our previous study demonstrated that the proportions of Th17 cells in the peripheral blood of AIT patients and the expression levels of its effector cytokine, IL-17A, are significantly increased, which is correlated with disease severity [4, 5], suggesting that Th17 cells may be involved in the autoimmune injury of AIT. Notch signaling is an evolutionarily highly conserved intercellular pathway, and it is associated with cell differentiation, cell proliferation, cell apoptosis, and epithelial-mesenchymal transition [6]. Recently, studies have reported that the Notch signaling pathway acts on ROR*γ*t, the specific transcription factor of Th17 cells, and further regulates Th17 cell differentiation. Therefore, the Notch signaling pathway plays an important role in many immune diseases, such as psoriasis and rheumatoid arthritis [7–9]. In the present study, we established an experimental autoimmune thyroiditis (EAT) mouse model to explore the potential role of the Notch signaling pathway in the thyroid autoimmune injury mediated by Th17 cells.

## 2. Materials and Methods

### 2.1. Experimental Mice

Female C57BL/6 mice aged 6 to 8 weeks weighing 15 to 18 g each were obtained from the Institute for Metabolic and Neuropsychiatric Disorders at Binzhou Medical University. The mice were reared under specific-pathogen-free- (SPF-) grade sterile conditions in the animal center of Binzhou Medical University Hospital. The mice were kept in a controlled environment at 23°C, 55% humidity, and 12 h/12 h light-dark cycle, and the experiment was initiated after 1 week of adaptive feeding. All animal experiments were approved by the Animal Experiment Ethics Committee of Binzhou Medical University Hospital and were conducted in accordance with the animal experimentation guidelines. Experimental mice were randomly divided into a control group (NC, *n* = 10) and an EAT group (*n* = 30). The EAT group was further divided into an EAT-A group (*n* = 20) and an EAT-B group (*n* = 10). Porcine thyroid immunoglobulin (pTg) (Sigma, USA) was dissolved in PBS and prepared for a mother liquor (2 mg/ml). Complete Freund's adjuvant (CFA) (Sigma) and incomplete Freund's adjuvant (IFA) (Sigma) were added into the mother liquor to prepare the primary and secondary immunization preparations (1 mg/ml) for use right after preparation. Mice in the EAT-A group were given multiple subcutaneous injections, including 100 *μ*l of the primary immunization preparation at week 1 and 100 *μ*l of the secondary immunization preparation at weeks 2 to 8. Mice in the EAT-B group were given an intraperitoneal injection of DAPT (10 mg/kg, Sigma) 30 min before the subcutaneous injection of the immunization preparation. Mice in the NC group were injected at the same sites with equal amounts of PBS (Sangon Biotech, China) via the same route. Mice from each group were anesthetized and sacrificed at week 8 of the experiment.

### 2.2. Histopathological Examination of Murine Thyroid

A midline incision of the mouse neck skin was made to expose the trachea. After the thyroid glands were harvested, they were fixed in 4% paraformaldehyde, embedded in paraffin, and sectioned and stained with hematoxylin and eosin (HE). Inflammatory severity was scored according to the degree of lymphocytic infiltration [10] as follows: 0, normal; 1+, 1-10%; 2+, 10-30%; 3+, 30-50%; 4+, >50%.

For the immunohistochemical (IHC) analyses of murine thyroid, paraffin sections of thyroid tissue were prepared and dewaxed, and the sections were immersed in antigen repair solution (Biosharp, China) on high heat for 10 min. After cooling the sections to room temperature, endogenous peroxidase blocker (ZSGB-BIO, China) was added dropwise to the sections and incubated for 10 min. The sections were then incubated overnight in 4°C with CD4 rabbit monoclonal antibody (mAb) (1 : 200, Cell Signaling Technology, USA), retinoic acid-related orphan receptor gamma (ROR*γ*) rabbit mAb (1 : 250, Abcam, UK), and IL-17A rabbit mAb (1 : 100, Cell Signaling Technology). The sections were then incubated with reaction enhancer solution (ZSGB-BIO) for 20 min at room temperature followed by incubation with goat anti-rabbit IgG antibodies (ZSGB-BIO) at room temperature for 20 min. Finally, DAB (ZSGB-BIO) was used to visualize the immunoreaction after counterstaining with hematoxylin.

### 2.3. ELISA for Serum TgAb Titer

Blood samples were collected from the heart. After standing for 30 min at room temperature, the serum was separated by centrifugation at 3000 rpm for 20 min. Subsequently, TgAb was measured using the mouse TgAb ELISA kit (Milbio, China) according to the manufacturer's instructions, and the TgAb titers were calculated according to the optical density (OD) at 450 nm.

### 2.4. Spleen Index

The body weight and spleen weight of the mice were recorded, and the spleen index was calculated according to the following formula: spleen index (mg/g) = spleen weight/body weight.

### 2.5. Preparation of Spleen Mononuclear Cell (SMC) Suspension

SMCs were isolated using the mouse SMC isolation kit (Tbdscience, China) according to the manufacturer's instructions, and SMCs were suspended in RPMI 1640 medium containing fetal bovine serum (FBS), penicillin/streptomycin, sulfhydryl reductant, and nonessential amino acid solution (Solarbio, China). The SMC suspensions were counted and adjusted to 10^6^ cells/ml.

### 2.6. Th17 Cell Culture, Polarization, and DAPT Treatment

Approximately 10^5^ SMCs from EAT-A mice were cultured in 12-well plates coated with CD3 mAb and CD28 mAb (Bioxcell, USA). The polarization of Th17 cells was stimulated by adding transforming growth factor-*β*1 (TGF-*β*1), recombinant interleukin-1*β* (rIL-1*β*), rIL-6, rIL-23 (Biolegend, USA), interferon-*γ* (IFN-*γ*) mAb, and IL-4 mAb (Bioxcell, USA) into each well. DAPT dissolved in DMSO (80 mmol/L) was added to the SMC suspensions, and the final concentrations of DAPT were adjusted to 10 *μ*mol/L, 20 *μ*mol/L, and 40 *μ*mol/L in RPMI 1640 medium. Moreover, the wells containing only an equivalent concentration of DMSO (equivalent to 0 *μ*mol/L DAPT) were used as the control group. Cells were cultured at 37°C and 5% CO_2_.

### 2.7. Preparation of Thyroid Mononuclear Cell (TMC) Suspension

The thyroid tissues were immersed in Dispase II (Sigma) at 37°C for 1 h and washed three times with PBS containing 1% penicillin/streptomycin. The tissues were then digested with sufficient digestive working fluid containing collagenase P (Sigma), DNase I (Sigma), and RPMI 1640 medium at 37°C for 2 h. The digestion was neutralized with RPMI 1640 medium containing 10% FBS. The TMC suspensions were passed through a 70 *μ*m cell filter, centrifuged at 1000 g for 5 min, and washed three times with PBS. Finally, the TMC suspensions were added into a 12-well plate (approximately 10^5^ cells/well).

### 2.8. Flow Cytometric Analysis of the Percentages of Th17 Cells

Phorbol 12*-*myristate 13-acetate (PMA, Solarbio) and Ca-ionomycin (Solarbio) were added into the 12-well plates containing the SMC or TMC suspension, and brefeldin A (BFA, Solarbio) was added after 1 h followed by incubation for 3 h (total stimulation of 4 h). Cells were then collected, washed, and surface stained with APC-labeled CD4 antibody (Biolegend) at 4°C in the dark for 30 min. IC fixation buffer (eBioscience, USA) was then added followed by incubation at 4°C in the dark for 20 min. Finally, cells were washed, resuspended in permeabilization buffer (eBioscience), and stained with PE-labeled IL-17A antibody (Biolegend) at 4°C in the dark for 30 min. Flow cytometric analyses were performed using a FACScanto flow cytometer (BD, USA).

### 2.9. Quantitative Real-Time PCR Analyses of Notch1, Hairy and Enhancer of the Split 1(Hes1), ROR*γ*t, and IL-17A mRNA

Total RNA of mice spleen cells was extracted with Trizol reagent (Invitrogen, USA), and complementary DNA (cDNA) was synthesized using the PrimeScript™RT reagent Kit (TaKaRa, Japan). The primers for Notch1, Hes1, ROR*γ*t, and IL-17A were designed and synthesized, and the target genes were detected using the CFX96 Touch™ Real-Time PCR Detection System (Bio-Rad, USA) with *β*-Actin as the internal reference. The thermocycler program was as follows: predenaturation at 95°C for 5 min, 40 cycles of denaturation at 95°C for 10 s, and annealing at 60°C for 60 s. The primer sequences are shown in Table 1.

### 2.10. Analysis of IL-17A Concentration in SMC Culture Supernatant by ELISA

SMC culture supernatants were collected, and the concentrations of IL-17A were measured using the mouse IL-17A ELISA kit (R&D Systems, USA) according to the manufacturer's instructions.

## 3. Statistical Analyses

According to the results of the normal distribution test (Shapiro-Wilk test), data are expressed as the mean ± standard deviation (SD). One-way analysis of variance (ANOVA), Welch's ANOVA, least-significant difference (LSD) test, Tamhane's T2 test, and Pearson's correlation coefficients were used for statistical analyses. All tests were completed by SPSS 24.0 and GraphPad Prism 8.0 software. *P* values < 0.05 were considered statistically significant.

## 4. Results

### 4.1. Histopathological Changes in the Thyroid

**Figure 1:**
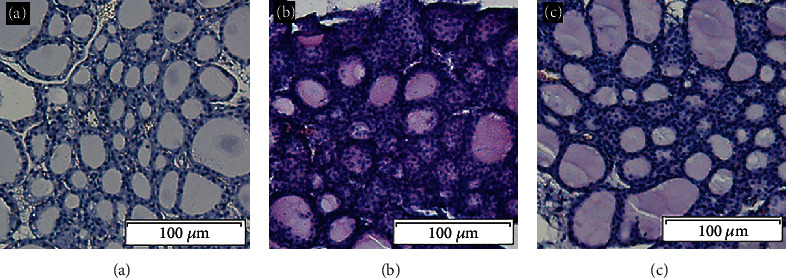
Representative thyroid sections from different groups (HE × 200). (a) The NC group had a severity score of 0. (b) The EAT-A group had a thyroiditis score of 4+. (c) The EAT-B group had a thyroiditis score of 2+.

Microscopy analysis indicated that the thyroid follicular epithelial cells of mice in the NC group were cuboidal and arranged in a single layer. In the thyroid glands of EAT mice, however, follicular epithelial cells were damaged, and different degrees of lymphocyte infiltration were observed. In the EAT-A group, the thyroid follicles showed obvious damage and atrophy with a large number of infiltrated lymphocytes. Interestingly, the degree of follicular destruction, atrophy, and lymphocyte infiltration in the EAT-B group was significantly reduced compared to the EAT-A group (Figure 1).

**Figure 2:**
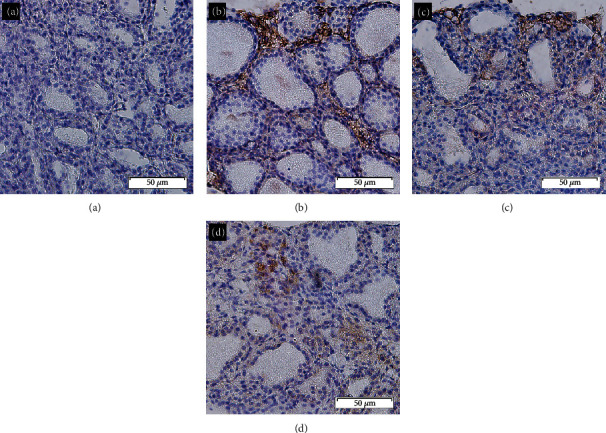
IHC (×200). (a) Negative control thyroid section. (b) CD4 positivity was found in the inflammatory cells around thyroid follicles. (c) ROR*γ* positivity was found in the infiltrated lymphocytes. (d) IL-17A expression was detected in the infiltrated lymphocytes, especially in the areas near the damaged thyroid follicles.

In EAT mice, the expression of CD4, ROR*γ*, and IL-17A was detected in the areas of infiltrating lymphocytes in the thyroid sections by IHC (Figure 2), which indicated that Th17 cells infiltrated the thyroid.

### 4.2. Serum TgAb Titers

**Figure 3:**
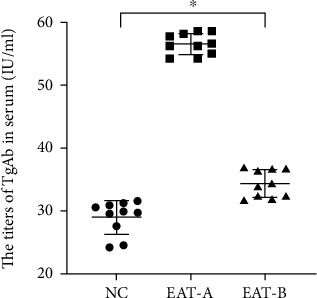
Serum TgAb titers. There were significant differences among the three groups (*F* = 435.793, ^∗^*P* < 0.001, one-way ANOVA). Interestingly, the titers in EAT-B mice were significantly decreased compared to EAT-A mice (*P* < 0.001, LSD test) but significantly higher compared to NC mice (*P* < 0.001, LSD test).

Compared to the NC group (28.96 ± 2.67 IU/ml) and EAT-B group (34.35 ± 2.19 IU/ml), the serum TgAb titers were significantly increased in the EAT-A group (56.57 ± 1.69 IU/ml), and the serum TgAb titers in the EAT-B group were significantly higher than those in the NC group (Figure 3).

### 4.3. Spleen Index

**Figure 4:**
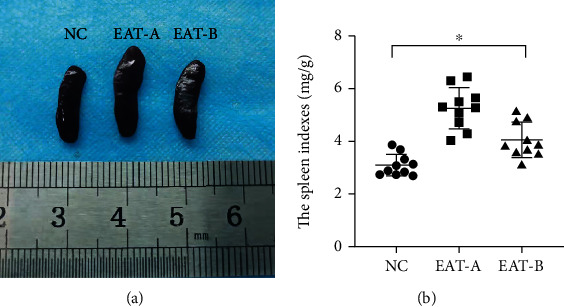
Spleen size and spleen index of mice at the end of the experiment. (a) Comparison of spleen size among the three different groups. (b) Comparison of the spleen index among the three groups (*F* = 28.703, ^∗^*P* < 0.001, one-way ANOVA). Similar to the spleen size, the spleen index in EAT-A mice was significantly increased compared to NC mice (5.26 ± 0.78 mg/g vs. 3.10 ± 0.41 mg/g, *P* < 0.001, LSD test), and EAT-B mice had a lower spleen index (4.07 ± 0.67 mg/g) than EAT-A mice (*P* < 0.001, LSD test) but higher than that of NC mice (*P* = 0.002, LSD test).

The spleen size of EAT-A mice was significantly enlarged, and the degree of splenomegaly in EAT-B mice was significantly reduced after blockade of the Notch signaling pathway (Figure 4(a)). The spleen index among the three groups had the same trend as the spleen size (Figure 4(b)).

### 4.4. Percentage of Th17 Cells in SMCs and TMCs

**Figure 5:**
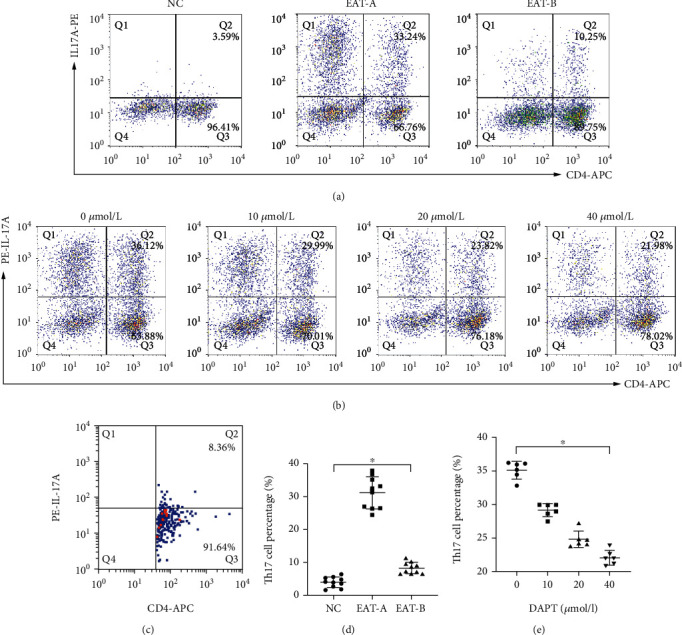
Flow cytometric analyses of Th17 cells in SMCs and TMCs. (a) Representative flow cytometry results of Th17 cells in SMCs in the three groups. (b) Representative flow cytometry results of Th17 cells in SMCs from EAT-A mice treated with different concentrations of DAPT in vitro. (c) Representative flow cytometry analyses of Th17 cells in TMCs from EAT-A mouse thyroids. (d) Summary of Th17 cell proportions. There were significant differences of Th17 cells in SMCs among the three groups (Welch *F* = 140.301, ^∗^*P* < 0.001, Welch's ANOVA). Additionally, Th17 cell percentages in EAT-A mice were significantly higher compared to those in NC mice (31.16 ± 4.79% vs. 3.99 ± 1.59%, *P* < 0.001, Tamhane's T2 test). In EAT-B mice, DAPT treatment via intraperitoneal injection significantly decreased the percentage of Th17 cells (7.76 ± 1.58%) compared to EAT-A mice (*P* < 0.001, Tamhane's T2 test). (e). Summary of Th17 cell percentages in four groups with different DAPT concentrations in vitro. Analyses revealed that Th17 cell percentages decreased in a concentration- dependent manner (*F* = 144.293, ^∗^*P* < 0.001, one-way ANOVA).

The percentages of Th17 cells in SMCs (Figures 5(a) and 5(b)) and TMCs (Figure 5(c)) were analyzed and are summarized in Figures 5(d) and 5(e). The Th17 cell proportions in EAT-A SMCs were significantly higher compared to NC SMCs, and they significantly decreased after DAPT treatment both in vivo and in vitro. In addition, the proportions of Th17 cells decreased in a dose-dependent manner with the concentration of DAPT in vitro. Moreover, a certain proportion of Th17 cells was detected in TMCs from EAT-A mice as indicated by IHC, which further confirmed the infiltration of Th17 cells in the thyroid gland (Figure 5(c)).

### 4.5. Expression of Notch1, Hes1, ROR*γ*t, and IL-17A mRNA and Correlation Analyses of All Genes

**Figure 6:**
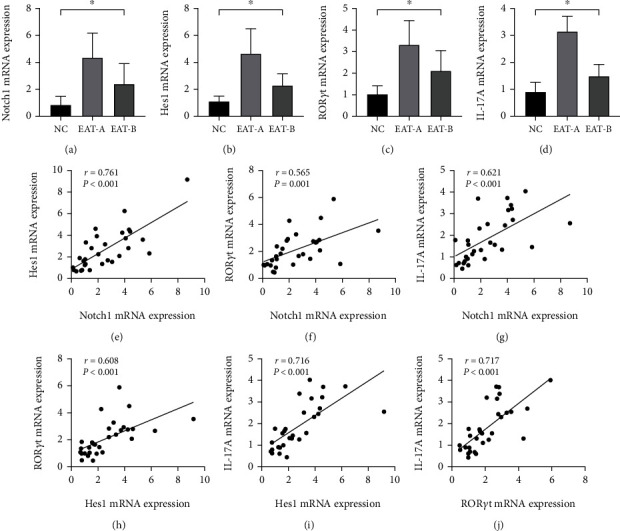
The Notch1, Hes1, ROR*γ*t, and IL-17A mRNA expression levels and correlation analyses of all genes (*n* = 30). One-way ANOVA analyses revealed that the mRNA expression levels of Notch1, Hes1, ROR*γ*t, and IL-17A were significantly different among the three groups (*F* = 15.186, 22.909, 17.538, and 63.968 respectively, all ^∗^*P* < 0.001, (a–d)). (a) Altered expression of Notch1 mRNA (NC: 0.83 ± 0.65, EAT-A: 4.33 ± 1.84, and EAT-B: 2.38 ± 1.51). EAT-B mice had significantly lower Notch1 mRNA levels than EAT-A mice (*P* = 0.005). (b) Altered expression of Hes1 mRNA (NC: 1.08 ± 0.42, EAT-A: 4.65 ± 1.85, and EAT-B: 2.31 ± 0.83). EAT-B mice had significantly decreased Hes1 mRNA expression compared to EAT-A mice (*P* < 0.001). (c) Altered expression of ROR*γ*t mRNA (NC: 1.01 ± 0.40, EAT-A: 3.32 ± 1.12, and EAT-B: 2.10 ± 0.94). Compared to EAT-A mice, the ROR*γ*t mRNA expression was significantly downregulated in EAT-B mice (*P* = 0.004). (d) Altered expression of IL-17A mRNA (NC: 0.90 ± 0.36, EAT-A: 3.15 ± 0.57, and EAT-B: 1.49 ± 0.43). IL-17A mRNA expression was significantly downregulated in EAT-B mice compared to EAT-A mice (*P* < 0.001). Correlation analyses revealed that the expression of the upstream receptor, Notch1, and the downstream target gene, Hes1, of the Notch signaling pathway positively correlated with ROR*γ*t and IL-17A mRNA expression, respectively (all *P* < 0.01, (e–j)).

The mRNA expression levels of the main components of the Notch signaling pathway (Notch1 and Hes1), the key transcription factor (ROR*γ*t), and the effector cytokine (IL-17A) of Th17 cells had the same trend as the proportions of Th17 cells among the three groups. Of note, the *γ*-secretase inhibitor, DAPT, inhibited the Notch signaling pathway and significantly downregulated the expression of the key related factors for the development and function of Th17 cells. Moreover, Notch1 and Hes1 mRNA expression levels were positively correlated with the mRNA expression levels of ROR*γ*t and IL-17A, which may indicate a close relationship between the Notch signaling pathway and Th17 cells (Figure 6).

### 4.6. IL-17A Concentrations in SMC Culture Supernatant

**Figure 7:**
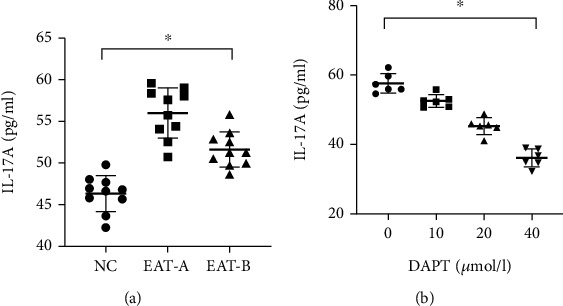
IL-17A concentrations in SMC culture supernatants in vivo and in vitro. (a) Altered levels of IL-17A in SMC culture supernatants. There were significant differences among the three groups (*F* = 38.882, ^∗^*P* < 0.001, one-way ANOVA). EAT-B mice had lower IL-17A concentrations than EAT-A mice (51.62 ± 2.13 pg/ml vs. 56.02 ± 3.00 pg/ml, *P* < 0.001), and both EAT-A and EAT-B mice had higher IL-17A concentrations than NC mice (46.32 ± 2.16 pg/ml, both *P* < 0.001). (b) Dose-dependent alterations of IL-17A levels in SMC culture supernatants after treatment with different concentrations of DAPT (*F* = 86.750, ^∗^*P* < 0.001, one-way ANOVA).

In EAT-A mice, the concentrations of IL-17A in the SMC culture supernatant were higher than those in NC mice (*P* < 0.001, Figure 7(a)). DAPT treatment significantly reduced IL-17A levels both in vivo and in vitro. Moreover, the IL-17A levels in the SMC supernatant from EAT-A mice were significantly decreased by different concentrations of DAPT in a dose-dependent manner (*P* < 0.001, Figure 7(b)).

### 4.7. Correlation Analyses between Th17 Cells and Other Experimental Indexes

**Figure 8:**
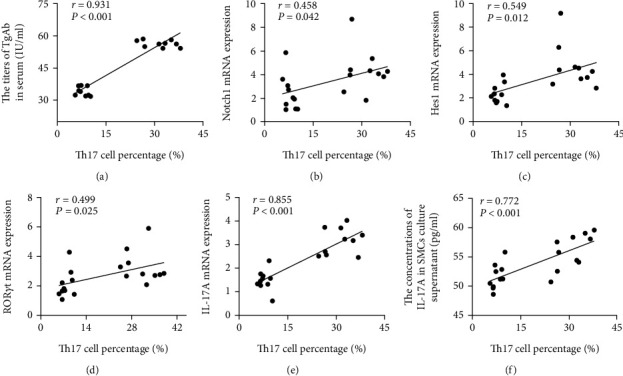
Correlation analyses of Th17 cells with TgAb and Notch signaling pathway-related components in EAT-A mice (*n* = 20). Th17 cell percentages positively correlated with (a) serum TgAb titers (*r* = 0.931, *P* < 0.001), (b) Notch1 mRNA expression (*r* = 0.458, *P* = 0.042), (c) Hes1 mRNA expression (*r* = 0.549, *P* = 0.012), (d) ROR*γ*t mRNA expression (*r* = 0.499, *P* = 0.025), (e) IL-17A mRNA expression (*r* = 0.855, *P* < 0.001), and (f) IL-17A concentration in SMC culture supernatant (*r* = 0.772, *P* < 0.001).

Correlation analyses indicated positive correlations of Th17 cells from EAT-A mice with serum TgAb titers (reflecting the degree of autoimmune injury of thyroid), Notch1 mRNA expression (upstream receptor of the Notch signaling pathway), Hes1 mRNA expression (downstream target gene of Notch signaling pathway), ROR*γ*t mRNA expression (key transcription factor), and IL-17A mRNA expression and supernatant concentration (effector cytokine of Th17 cells) (all *P* < 0.05; Figure 8).

## 5. Discussion

CD4^+^ T lymphocytes play a critical role in the immune response and inflammatory diseases. Naive CD4^+^ T lymphocytes are activated by antigen-presenting cells (APCs), CD28 and other costimulatory molecules [11], and they differentiate into Th17 cells under the combined induction of IL-6, IL-1*β*, IL-23, and TGF-*β* [12, 13]. Moreover, this subset of CD4^+^ T cells acts on target cells and plays critical roles in immune inflammatory response by mainly secreting the IL-17A proinflammatory cytokine. The ROR*γ*t has been identified as the key transcription factor for the development of Th17 cells, and it guides the secretion of IL-17A [14, 15]. It has been demonstrated that Th17 cells and the expression of their effector cytokine, IL-17A, are increased in autoimmune diseases, including psoriasis, multiple sclerosis, and rheumatoid arthritis [16–18]. Our previous study found that Th17 cells and IL-17A are also increased in Hashimoto's thyroiditis (HT) patients and positively correlated with the titers of the TPOAb and TgAb thyroid autoimmune injury markers [4, 5, 19]. These results suggest that Th17 cells may be involved in the occurrence and development of thyroid-specific autoimmune injury in HT patients. To further clarify the role of Th17 cells in AIT autoimmune injury, the present study confirmed that Th17 cells infiltrated the thyroid in EAT mice. Additionally, the percentages of Th17 cells in SMCs from EAT mice and the levels of their effector cytokine, IL-17A, were significantly increased in EAT mice. Moreover, the percentage of Th17 cells was positively correlated with TgAb titer and IL-17A content, which indicated that Th17 cells may be involved in the occurrence and development of thyroid autoimmune injury in EAT mice and that the proinflammatory effect of Th17 cells may be enhanced by secreting IL-17A.

Notch is a highly conserved transmembrane protein. After the receptor binds with two kinds of membrane-bound ligands, namely, Jagged (Jagged1 and Jagged2) and Delta-like (DLL-1, DLL-3, DLL-4, japped-1, and japped-2), the Notch1 signaling pathway is activated. Notch intracellular domain (NICD) is formed by *γ*-secretase proteolysis in the transmembrane region, which initiates its downstream transcription factor, Hes1, and induces a biological effect [20–22]. The DAPT *γ*-secretase inhibitor acts on the presenilin fragment, which is the catalytic component of *γ*-secretase, and then blocks the production of NICD [23, 24]. Our previous study found that Notch1 is highly expressed in PBMCs from HT patients and is positively correlated with TPOAb and TgAb titers [19]. The present study further confirmed that Notch1 and its target gene, Hes1, were highly expressed in EAT mice. In addition, the expression levels of Notch1 and Hes1 were downregulated after DAPT treatment to inhibit *γ*-secretase in vivo, which was accompanied by significant alleviation of lymphocyte infiltration, follicular atrophy, and destruction degree in mice thyroid tissues. Together, these findings suggested that the Notch signaling pathway may be closely related to thyroid autoimmune injury.

The Notch signaling pathway plays an important role in the proliferation and differentiation of early T lymphocytes and the functional regulation of mature T lymphocytes [25–27]. Additionally, the expression of the Notch ligands Jagged1 and DLL-4 on APCs promotes the differentiation of Th17 cells [28, 29], and IL-17 and ROR*γ*t are direct transcriptional targets of the Notch signaling pathway in Th17 cells [7]. It has been reported that the Notch signaling pathway is blocked by DAPT treatment in some inflammatory disease models, such as asthma, autoimmune encephalomyelitis, and psoriasis, which is accompanied by decreased Th17 cell percentage and IL-17A level as well as significant improvement in disease severity [8, 23, 30, 31]. As mentioned earlier, the present study demonstrated that Notch signaling was activated in the cytokine environment promoting Th17 differentiation. However, inhibition of the Notch signaling pathway in vivo significantly downregulated the expression of the Notch1 signaling molecule and its target genes, Hes1, in EAT mice. Blockade of Notch signaling also decreased the Th17 cell proportion, the expression of the ROR*γ*t transcription factor and the secretion of the IL-17A effector cytokine in EAT mice. Similarly, the treatment of DAPT in vitro decreased the proportion of Th17 cells and the secretion of IL-17A in a dose-dependent manner in EAT mouse SMCs. In addition, there were positive correlations of the Th17 cell proportion with Notch signaling pathway-related expression levels (Notch1 and Hes1) as well as with ROR*γ*t and IL-17A, which revealed that the changing trend of the Notch signaling pathway was consistent with that of Th17 cells. Therefore, these results further suggested that the Notch signaling pathway may participate in the initiation and progression of thyroid autoimmune injury in EAT mice by promoting the differentiation and function of Th17 cells.

## 6. Conclusions

Taken together, the present findings suggested that the Notch signaling pathway may participate in thyroid autoimmune injury by promoting the differentiation of Th17 cells and enhancing their inflammatory effect. However, additional in-depth studies on the precise pathogenesis of AIT are necessary to provide new strategies and directions for the etiological treatment of AIT.

## Figures and Tables

**Table 1:** List of primers for qRT-PCR.

Primers		Sequence (5′→3′)
Notch1	Forward	TGCCTTGAGTGTGCTGGAATG
	Reverse	ATTCTGCCACAGGCGTATACTTGA

Hes1	Forward	AAAGACGGCCTCTGAGCAC
	Reverse	GGTGCTTCACAGTCATTTCCA

ROR*γ*t	Forward	TCTGCAAGACTCATCGACAAGG
	Reverse	CACATGTTGGCTGCACAGG

IL-17A	Forward	GGAAAGCTGGACCACCACA
	Reverse	CACACCCACCAGCATCTTCTC

*β*-Actin	Forward	AGTTGCGTTACACCCTTTCTTG
	Reverse	TCACCTTCACCGTTCCAGTTT

## Data Availability

The data used to support the findings of this study are available from the corresponding authors upon request.
